# Associations Between *CAMKK1* Polymorphism rs7214723 and the Prognosis of Patients With Lung Cancer

**DOI:** 10.3389/fonc.2021.757484

**Published:** 2021-11-19

**Authors:** Haorui Zhang, Bocen Chen, Zixiu Zou, Jian Feng, Yutao Li, Yi Wang, Xing He, Chang Xu, Haijian Wang, Shicheng Guo, Li Jin, Qiang Li, Jiucun Wang, Man Xiao, Feng Li, Junjie Wu

**Affiliations:** ^1^ Department of Ophthalmology, Changhai Hospital, Navy Military Medical University, Shanghai, China; ^2^ Department of Biochemistry and Molecular Biology, Hainan Medical University, Haikou, China; ^3^ State Key Laboratory of Genetic Engineering, Collaborative Innovation Center for Genetics and Development, School of Life Sciences, Fudan University, Shanghai, China; ^4^ Department of Thoracic Surgery, Shanghai Chest Hospital, Shanghai Jiaotong University, Shanghai, China; ^5^ Department of Urology, Navy Military Medical University Affiliated Changhai Hospital, Shanghai, China; ^6^ Clinical College, Xiangnan University, Chenzhou, China; ^7^ Department of Medical Genetics, School of Medicine and Public Health, University of Wisconsin-Madison, Madison, WI, United States; ^8^ Human Phenome Institute, Fudan University, Shanghai, China; ^9^ Department of Respiratory and Critical Care Medicine, Shanghai East Hospital, Tongji University, Shanghai, China; ^10^ Department of Respiratory Disease, Shanghai Public Health Clinical Center, Shanghai, China; ^11^ Department of Infectious Diseases, Renji Hospital, School of Medicine, Shanghai Jiao Tong University, Shanghai, China; ^12^ Department of Respiratory and Critical Care Medicine, Changhai Hospital, Navy Military Medical University, Shanghai, China

**Keywords:** *CAMKK1*, rs7214723, single nucleotide polymorphism, lung cancer, prognosis

## Abstract

**Background:**

The 5-year survival rate of patients with lung cancer in China is less than 20% and predicting their prognosis is challenging. We investigated the association between a common non-synonymous single nucleotide polymorphism (SNP), rs7214723, in the Ca^2+^/calmodulin-dependent protein kinase kinase 1 (*CAMKK1*) gene and the prognosis of patients with lung cancer.

**Methods:**

Genomic DNA was extracted from the blood samples of 839 patients with lung cancer, recruited from Changhai Hospital (n = 536) and Taizhou Institute of Health Sciences (n = 352), and genotyped using the SNPscan technique. The association between patient prognosis and the genotypic data for *CAMKK1* was analyzed using a multivariate Cox proportional hazards model adjusted for multiple potential confounders. The CRISPR/Cas9 gene-editing system was used to introduce point mutations in the *CAMKK1* rs7214723 of A549 and NCI-H358 cells. Subsequently, Cell proliferation and migration ability were assessed with the Cell Counting Kit-8 and scratch assay. The Annexin V-FITC apoptosis detection kit was used to detect cell apoptosis.

**Results:**

The *CAMKK1* rs7214723 recessive CC genotype conferred significantly better overall survival (CC *vs*. TT + TC: adjusted hazard ratio = 0.78, 95% confidence interval [CI], 0.61-1.00, *P* = 0.049) than the TT + TC genotypes. Stratified analysis showed that the *CAMKK1* rs7214723 CC genotype and recessive CC genotype conferred a significantly decreased risk of death in patients who were male, had a smoking history, or had stage III + IV cancer, compared with the TT and TT + TC genotypes. Relative to the TT + TC genotypes, the rs7214723 recessive CC genotype was also associated with a decreased risk of death in patients aged < 60 years (CC *vs*. TT + TC: adjusted hazard ratio = 0.59, 95% CI, 0.37-0.93, *P* = 0.024) and patients with squamous cell carcinoma (CC *vs*. TT + TC: adjusted hazard ratio = 0.65, 95% CI, 0.44-0.98, *P* = 0.038). Remarkably, CRISPR/Cas9-guided single nucleotide editing demonstrated that *CAMKK1* rs7214723 T > C mutation significantly inhibits cell proliferation and migration and promotes cell apoptosis.

**Conclusions:**

*CAMKK1* SNP rs7214723 may be a significant prognostic factor for the risk of death among patients with lung cancer.

## Introduction

Despite significant research efforts in the diagnosis and treatment of lung cancer in recent decades, reliable prediction of prognosis remains challenging. The 5-year survival rate of patients with lung cancer remains lower than 20% (1, 2). Although the most important prognostic determinant of lung cancer is the tumor, lymph node, and metastasis (TNM) staging system, the wide range of survival rates among patients with non-small cell lung cancer with the same stage indicates considerable heterogeneity of tumors that possibly differ in biology, which consequently confers different prognoses (3, 4). Smoking is the most significant factor associated with the risk and prognosis of lung cancer. Approximately 80%-90% of lung cancer cases are caused by smoking. However, only 15% of smokers develop lung cancer (5, 6). The other known risk factors for lung cancer include environmental factors such as air pollution and occupational exposure to carcinogens (7, 8). Moreover, the main reasons for lung cancer development are genetic and epigenetic damage caused by environmental carcinogens (7). Increasing evidence reveals that both the risk and prognosis of lung cancer are influenced by the interaction between environmental risk factors and individual genetic factors (6, 9). Therefore, establishing accurate prognostic indicators is crucial for improving survival among patients with lung cancer, whose prognosis depends on several factors.

The Ca^2+^/calmodulin-dependent protein kinases (CaMKs) family member CaMK kinase (CaMKK) has two subtypes: CaMKK1 and CaMKK2 (10, 11). Increased Ca^2+^ concentration enhances the activity of CaMKK, which phosphorylates and activates specific downstream protein kinases, including Ca^2+^/calmodulin-dependent protein kinases I (CaMK I), CaMK IV, and 5’-AMP-activated protein kinase (AMPK), which subsequently mediate multiple Ca^2+^ signaling cascades (12–14). These cascade reactions are not only involved in the development of neurons (15–17) and the regulation of mitochondrial morphology (18); they also regulate transcriptional activation through phosphorylation of cAMP-response element-binding proteins and serum response factors (19–21). It also affects transcriptional activation of glycolytic genes (22). Inhibiting the activity of CaMK/CaMKK can also promote cell apoptosis (12, 23, 24). Williams et al. (25) have demonstrated that the activation of CaMK II and CaMK IV inhibits cell cycle progression in small cell lung cancer (SCLC) cells. In addition, Hülsmann et al. (26) determined that AMPK activation contributes to the inhibition and apoptosis of growing human lung cancer cells. Therefore, we hypothesize that *CAMKK1* may also play an essential role in the development of lung cancer.

Single nucleotide polymorphisms (SNPs) is a common form of genetic variation in humans. A large number of studies have explored the effects of multiple lung cancer-related gene polymorphisms on the treatment efficacy and prognosis of patients with lung cancer (27–31). The findings of Rudd’s (32) and Chen’s (33) studies on 1529 British and 320 Chinese patients, respectively, with lung cancer showed that the SNP, rs7214723, in the *CAMKK1* gene can increase the risk of lung cancer. Furthermore, activation of the CaMKK/CaMK IV pathway in hepatocellular carcinoma (HCC) leads to increased secretion of high-mobility group box 1, which significantly promotes the progression of HCC and leads to poor prognosis; in contrast, CaMKK/CaMK IV inhibitors can significantly reduce the invasion and metastasis of HCC cells *in vitro* (34). To further investigate whether *CAMKK1* SNP rs7214723 is associated with lung cancer progression, we collected peripheral blood samples from 839 patients with a confirmed lung cancer diagnosis before treatment initiation and performed genotyping and patient follow-up. The association between *CAMKK1* SNP rs7214723 as well as the prognosis and survival of patients with lung cancer was evaluated. Moreover, to further demonstrate the effects of *CAMKK1* SNP rs7214723 on Cell viability and apoptosis, the CRISPR/Cas9 gene editing system was used to introduce point mutations in *CAMKK1* rs7214723 in A549 and NCI-H358 cells. Cell proliferation and migration were assessed with the Cell Counting Kit-8 and scratch assay, respectively. The annexin V-FITC apoptosis detection kit was used to detect cell apoptosis.

## Materials and Methods

### Patients

A total of 888 patients with primary lung cancer were enrolled from January to November 2009; subsequently, 49 patients were excluded because of incomplete data. Some members of our cohort were recruited from Changhai Hospital, which is affiliated to the Naval Military Medical University (Second Military Medical University) (n = 536); the others were recruited from the Taizhou Institute of Health Sciences, Fudan University (n = 352). The inclusion criterion was primary lung cancer diagnosis by histopathological examination, with no history of malignancy in other organs. No age or sex limitations were set in this study. Medical records were used to obtain patients’ clinical data, and follow-up data were collected through telephone interviews. The ethics committee of the School of Life Sciences, Fudan University approved this study. Informed consent was obtained from all study subjects.

### SNP Selection and Genotyping

Prior to treatment initiation, 5-mL blood was collected from each patient. The QIAamp DNA Blood Mini Kit (Qiagen, 51106) was used to extract genomic DNA, while a 2 × 48-plex SNPscan TM kit (Genesky Biotechnologies, G0104) was used for genotyping, as previously described (35, 36). SNPscan is a proprietary multiplex SNP genotyping system that allows for the simultaneous genotyping of 48, 96, 144, or 192 SNPs per sample—all using a single tube/sample. Moreover, SNPscan discriminates alleles through a highly specific ligation reaction. We determined the genotyping quality using a detailed procedure comprising a successful call rate set at > 95%, duplicate genotype calling, having samples of internal positive controls, and performing the Hardy–Weinberg equilibrium (HWE) test. In addition, the genotyping assays were performed by personnel who were blinded to the patients’ clinical information.

### Cell Lines, Media, and Cell Culture Conditions

The cancer cell lines, A549 (ATCC, CRM-CCL-185) and NCI-H358 (ATCC, CRL-5807), were employed for the *in vitro* assays and for the gene editing study. The cells were cultured in RPMI-1640 medium (Gibco, C22400500BT) supplemented with 10% fetal bovine serum (Hyclone, SH30084.03HI), penicillin (100 IU/mL), and streptomycin (50 μg/mL) at 37°C with 5% CO_2_. The medium renewal interval was obtained from the ATCC animal cell culture guide, downloaded from the ATCC website.

### Genome Editing Using CRISPR-Cas9

The sgRNA was designed using the CRISPR/Cas9 Design Tool (http://crispr.mit.edu), targeting the exon 12 of the *CAMKK1* gene. The designed primers were annealed and joined to form double-stranded DNA (dsDNA). The dsDNA was inserted *via* BbsI (NEB R0539S) and T4 DNA Ligase into the pSpCas9 (BB)-2A-Puro (PX459) V2.0 plasmid (Addgene 62988)—named PX459.*CAMKK1*. The A549 and NCI-H358 cells were lipofected (Lipo8000TM, Beyotime, C0533) with the PX459.*CAMKK1* plasmid and the designed ssDNA; the resulting cells were named A549 *CAMKK1*-W and NCI-H358 *CAMKK1*-W, respectively. Transfected cells were positively selected with puromycin dihydrochloride (Beyotime, ST551) 24 hours after lipofection. Positive clones were selected for polymerase chain reaction (PCR) and, subsequently, the PCR products were sequenced.

### Cell Viability Assays

Cell proliferation was assessed using the Cell Counting Kit-8 (SAB biotech, CP002-1) according to the manufacturer’s instructions. Cells were seeded in 96-well plates at a density of 1 × 10^3^ cells/well in 100 μL cell medium and cultured at 37°C with 5% CO_2_, 10 μL of CCK-8 solution was added to the cell culture after 24 hours. After 3–4 hours of incubation, cell proliferation was evaluated by spectrophotometry, using an Epoch microplate spectrophotometer (BioTek, USA), at an absorbance of 450 nm. The experiment was repeated three times. Cell migration was assessed with the scratch assay. About 5 × 10^3^ cells were seeded into a 6-well plate and cultured for 24 h. The cells were then scratched vertically with a pipette tip and then washed with phosphate-buffered saline (PBS) three times to remove the detached cells. Serum-free medium was added, and the cells were cultured at 37°C with 5% CO_2_. After 24 hours, samples were taken, photographs were acquired, and control wells were set for each group of experiments; the assay was repeated three times.

### Apoptosis Detection

The annexin V-FITC apoptosis detection kit (Beyotime, C1062S) was used to detect cell apoptosis. Cells were plated in six-well plates at a density of 5 × 10^5^ cells/well. The apoptosis rate was analyzed by flow cytometry after 24 h of incubation; control wells were set for each group of experiments, and the assay was repeated three times.

### Statistical Analysis

Pearson’s chi-square test was used to check for HWE. The overall survival was calculated from the sample collection date to the date of either death from any cause or the last follow-up visit. The Kaplan–Meier method was used to estimate the median survival time, and differences between groups were tested using the log-rank test. The hazard ratio (HR) and the 95% confidence interval (CI) with age, sex and hospital adjustments were estimated using multivariate Cox regression analysis. Four SNP genetic models (allele, genotype, dominant, and recessive) were analyzed. In addition, stratified analyses were performed according to age, sex, smoking status, malignant cancer family history, TNM stage, and lung cancer histologic type. An independent sample t-test was used for comparison between two groups of cells. The statistical significance was considered at *P* < 0.05. All respective tests were two-sided. Statistical analyses were performed using R version 3.6.2 (Vienna, Austria).

## Results

### Patient Demographics and Clinical Characteristics

**Figure 1:**
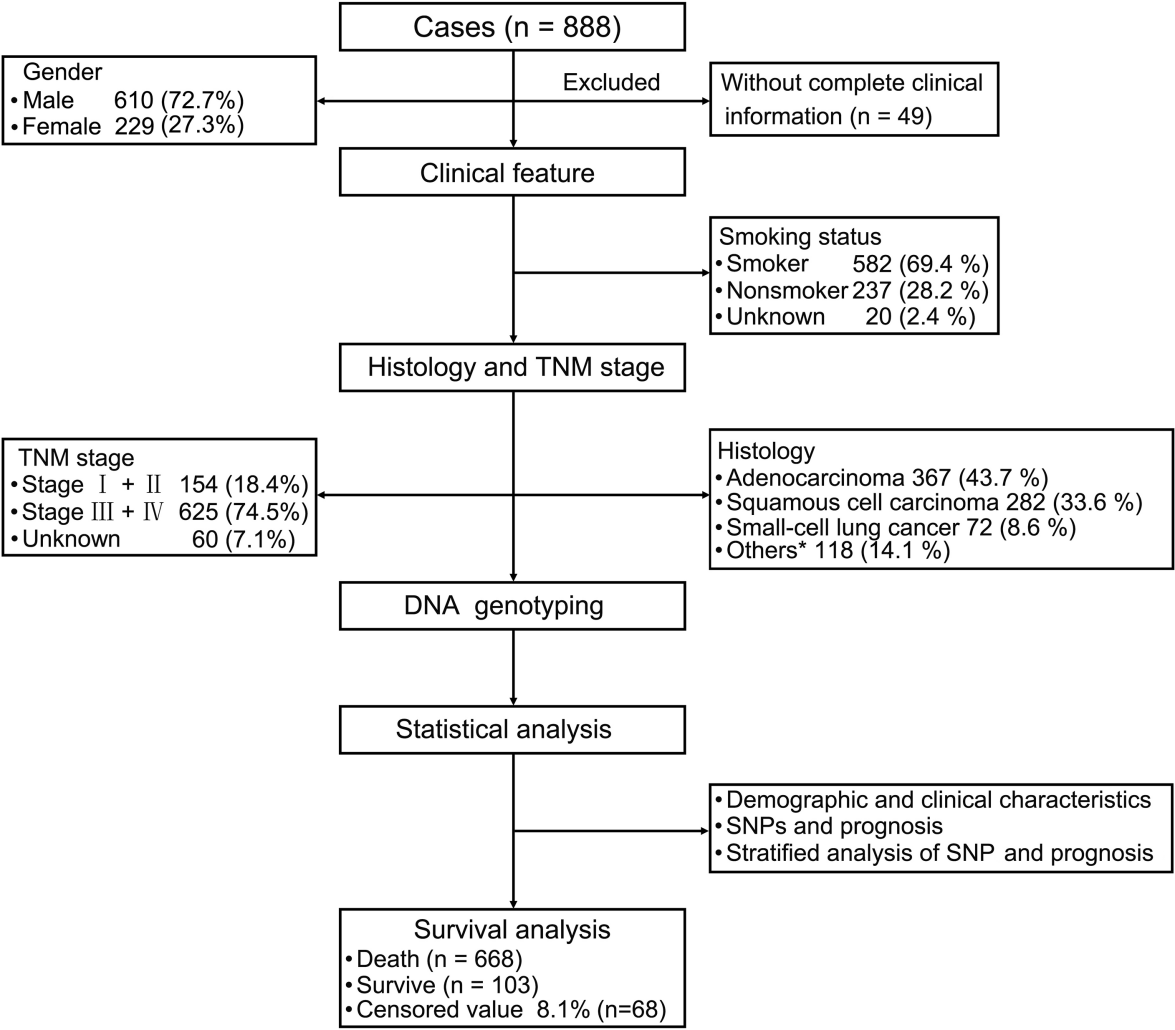
Patient demographics and clinical characteristics. We evaluated patients with lung cancer in terms of their clinical features and performed strategic analysis. For the 839 patients with primary lung cancer (excluding 49 patients because of insufficient clinical information), we evaluated their clinical features including smoking status, tumor-node-metastasis stage, and histology. Statistical analysis of demographic and clinical characteristics, single nucleotide polymorphisms (SNPs), and prognosis were performed. Stratified analysis of SNPs and prognosis were also performed. ^*^Other carcinomas include ASC, LCC, CS and MST.

The follow-up period was set from the start of enrollment until November 15, 2019. After excluding 49 patients because of incomplete clinical information, the data from 839 patients were analyzed. The study sample was an ethnically homogeneous group of Han Chinese individuals. A total of 668 (79.6%) patients died, 103 (12.3%) survived for longer than 5 years, and 68 (8.1%) were lost to follow-up. A total of 610 (72.7%) patients were male, 524 (62.5%) were aged ≥ 60 years, 582 (69.4%) had a history of smoking, and 302 (36%) had a family history of malignant cancer. As for the cancer subtypes, 367 (43.7%) patients were diagnosed with adenocarcinoma, 282 (33.6%) with squamous cell carcinoma (SCC), 72 (8.6%) with SCLC, and 118 (14.1%) with other cancer types, including adenosquamous carcinoma (ASC), large cell carcinoma, carcinosarcoma (CS) and mucoepidermoid carcinoma (MEC). There were 154 (18.4%) patients who were diagnosed with stage I and stage II disease, and 625 (74.5%) patients had stage III and stage IV disease (**Table 1** and **Figure 1**).

**Table 1:** Distribution of characteristics in Chinese patients with lung cancer and prognosis analysis.

Variables	N (%)	MST^#^	*P*
**All**	839	36.73	
**Gender**			0.01
Female	229 (27.3%)	40.17	
Male	610 (72.7%)	34.27	
**Age**			0.003
Age < 60	315 (37.5%)	40.87	
Age ≥ 60	524 (62.5%)	33.20	
**Smoking status**			< 0.001
Nonsmoker	237 (28.2%)	41.03	
Smoker	582 (69.4%)	33.90	
Unknown	20 (2.4%)	67	
**Family history of malignant cancer**			0.462
Yes	302 (36%)	33.63	
No	537 (64%)	38.03	
**Histology**			0.211
ADC	367 (43.7%)	38.80	
SCC	282 (33.6%)	33.63	
SCLC	72 (8.6%)	33.90	
Others*	118 (14.1%)	36.20	
**TNM Stage**			< 0.001
Stage I + II	154 (18.4%)	113.93	
Stage III + IV	625 (74.5%)	29.40	
Unknown	60 (7.1%)	66.43	

*Other carcinomas include ASC, LCC, CS and MEC. ^#^MST, median survival time.

### Association Between Patient Characteristics and Lung Cancer Outcomes

As shown in **Table 1**, the median survival time was significantly lower among males than females (34.27 *vs*. 40.17 months; *P* = 0.01), among patients aged ≥ 60 years than those aged < 60 years (33.2 *vs*. 40.87 months; *P* = 0.003), and among smokers than non-smokers (33.9 *vs*. 41.03 months; *P* < 0.001). Moreover, patients with advanced tumor stage had a significantly shorter median survival time than patients with early-stage tumors (29.4 *vs*. 113.93 months; *P* < 0.001) (**Table 1**). Association between patient characteristics and lung cancer outcomes in different hospitals showed no statistically significant difference (**Supplementary Table S1**).

### Association Between *CAMKK1* Polymorphism and Lung Cancer Prognosis

There were 341 TT, 400 TC, and 91 CC genotypes at *CAMKK1* rs7214723, with a genotype detection rate of 99.17% (**Table 2**). The genotype frequency at rs7214723 T/C of *CAMKK1* was consistent with HWE (*P* = 0.102), indicating that the investigated population was in a genetic balance (i.e., the population survey data were credible). Moreover, 1082 T alleles and 582 C alleles were detected at the rs7214723 locus of *CAMKK1*. The frequency of alleles T and C were 65.16% (864/1326) and 34.84% (462/1326), respectively, among the group of patients who died. Compared with the TT+TC genotypes, the risk of death was lower among individuals with the recessive CC genotype (adjusted HR for CC =0.78; 95% CI: 0.61-1.00, *P* = 0.049) (**Table 2**).

**Table 2:** Association between *CAMKK1* polymorphisms and prognosis in Chinese patients with lung cancer.

Model	Death/survive	HR (95% CI)*	*P*	HR^a^ (95% CI)	*P* ^a^
**Allele**					
T (ref)	864/218	1		1	
C	462/120	0.95 (0.85-1.07)	0.421	0.94 (0.84-1.05)	0.274
**Genotype**					
T/T (ref)	273/68	1		1	
T/C	318/82	1.06 (0.90-1.25)		1.04 (0.88-1.23)	0.624
C/C	72/19	0.82 (0.63-1.07)	0.149	0.80 (0.61-1.04)	0.093
**Dominate**					
T/T (ref)	273/68	1		1	
T/C+C/C	390/101	1.01 (0.86-1.18)	0.911	0.99 (0.84-1.16)	0.877
**Recessive**					
T/T+T/C (ref)	591/150	1		1	
C/C	72/19	0.80 (0.62-1.02)	0.075	0.78 (0.61-1.00)	0.049

*CI, confidence interval; HR, hazard ratio; ref, reference.

a Adjusted by age, sex and hospital.

### Association Between *CAMKK1* Polymorphism and Lung Cancer Prognosis Stratified by Patient Characteristics

We observed that among male patients, compared with genotype TT, genotype CC conferred a lower risk of death than genotype TT (*P* = 0.026) (**Table 3**), as did the recessive genotype CC relative to genotypes TT+TC (*P* = 0.024) (**Table 4**). In patients less than 60 years of age, the recessive genotype CC had a lower prognostic risk of death than genotypes TT+TC (*P* = 0.024). Among patients who were smokers, compared with genotype TT, genotype CC conferred a lower risk of death than genotype TT (*P* = 0.026) (**Table 3**), as did the recessive genotype CC compared with genotypes TT+TC (*P* = 0.028) (**Table 4**). For patients with SCC, the rs7214723 recessive genotype CC decreased the prognostic risk of death relative to TT+TC (*P* = 0.038) (**Table 4**). In patients with stage III + IV disease, the rs7214723 genotype CC had a lower prognostic risk of death than genotype TT (*P* = 0.049) (**Table 3**). The recessive genotype CC conferred a lower prognostic risk of death than genotypes TT+TC (*P* = 0.020) (**Table 4**).

**Table 3:** Association between *CAMKK1* SNP rs7214723 in genotype models and prognosis in Chinese patients with lung cancer.

Stratification	Death/survive			T/C VS T/T				C/C VS T/T			
	T/T (ref)	T/C	C/C	HR (95% CI)*	*P*	HR^a^ (95%CI)	*P* ^a^	HR (95%CI)	*P*	HR^a^ (95%CI)	*P* ^a^
**Gender**											
Male	200/40	239/51	58/16	0.96 (0.79-1.16)	0.666	0.97 (0.80-1.17)	0.731	0.70 (0.52-0.95)	0.020	0.71 (0.53-0.96)	0.026
Female	73/28	79/31	14/3	1.26 (0.91-1.73)	0.164	1.23 (0.89-1.70)	0.205	1.19 (0.67-2.13)	0.555	1.15 (0.64-2.06)	0.641
**Age (year)**											
≥ 60	182/32	201/46	50/9	0.99 (0.81-1.21)	0.948	1.01 (0.82-1.23)	0.936	0.87 (0.64-1.20)	0.400	0.89 (0.64-1.22)	0.454
< 60	91/36	117/36	22/10	1.21 (0.92-1.60)	0.169	1.15 (0.87-1.53)	0.330	0.71 (0.44-1.14)	0.155	0.64 (0.39-1.05)	0.076
**Smoking status**											
Yes	195/35	233/46	54/14	0.95 (0.78-1.15)	0.596	0.96 (0.79-1.16)	0.649	0.69 (0.51-0.94)	0.018	0.71 (0.52-0.96)	0.026
No	72/29	80/35	17/2	1.23 (0.89-1.70)	0.204	1.20 (0.87-1.66)	0.259	1.24 (0.73-2.11)	0.433	1.17 (0.68-2.01)	0.569
**Family history of malignant cancer**											
Yes	95/23	118/24	30/7	1.16 (0.88-1.52)	0.291	1.16 (0.88-1.52)	0.297	0.85 (0.56-1.28)	0.429	0.88 (0.58-1.33)	0.532
No	178/45	200/58	42/12	1.01 (0.83-1.24)	0.912	0.97 (0.79-1.18)	0.744	0.81 (0.58-1.14)	0.233	0.76 (0.54-1.07)	0.113
**Histology**											
ADC	120/38	126/43	28/8	1.10 (0.86-1.42)	0.440	1.00 (0.77-1.29)	0.996	1.01 (0.67-1.53)	0.952	0.97 (0.64-1.47)	0.893
SCC	95/18	112/17	29/8	1.15 (0.87-1.51)	0.317	1.16 (0.87-1.53)	0.312	0.73 (0.48-1.12)	0.149	0.71 (0.46-1.09)	0.115
**TNM stage**											
Stage I + II	34/31	33/37	8/9	0.93 (0.58-1.51)	0.777	0.81 (0.50-1.34)	0.418	1.01 (0.46-2.19)	0.982	1.05 (0.47-2.35)	0.909
Stage III + IV	222/34	259/41	55/9	1.08 (0.90-1.29)	0.410	1.07 (0.89-1.28)	0.470	0.75 (0.55-1.01)	0.058	0.74 (0.54-1.00)	0.049

*CI, confidence interval; HR, hazard ratio; ref, reference.

a Adjusted by age, sex and hospital.

**Table 4:** Association between *CAMKK1* polymorphisms in gene model and prognosis in Chinese patients with lung cancer.

Stratification	Allele model						Recessive model					
	Death/survive		HR (95% CI)*	*P*	HR^a^ (95% CI)	*P* ^a^	Death/survive		HR (95% CI)	*P*	HR^a^ (95% CI)	*P* ^a^
	T(ref)	C					T/T+T/C (ref)	C/C				
**Gender**												
Male	639/131	355/83	0.87 (0.77-0.99)	0.040	0.88 (0.77-1.00)	0.054	439/91	58/16	0.72 (0.55-0.95)	0.020	0.73 (0.55-0.96)	0.024
Female	225/87	107/37	1.15 (0.91-1.45)	0.246	1.13 (0.89-1.42)	0.315	152/59	14/3	1.06 (0.61-1.84)	0.838	1.03 (0.59-1.81)	0.913
**Age (year)**												
≥ 60	565/110	301/64	0.95 (0.83-1.10)	0.499	0.96 (0.84-1.11)	0.592	383/78	50/9	0.88 (0.65-1.18)	0.384	0.88 (0.65-1.19)	0.409
< 60	299/108	161/56	0.95 (0.78-1.15)	0.611	0.90 (0.74-1.10)	0.318	208/72	22/10	0.64 (0.41-1.00)	0.051	0.59 (0.37-0.93)	0.024
**Smoking status**												
Yes	623/116	341/74	0.87 (0.76-0.99)	0.037	0.88 (0.77-1.00)	0.054	428/81	54/14	0.71 (0.54-0.95)	0.021	0.72 (0.54-0.96)	0.028
No	224/93	114/39	1.15 (0.92-1.44)	0.231	1.12 (0.89-1.41)	0.332	152/64	17/2	1.11 (0.67-1.84)	0.679	1.06 (0.64-1.77)	0.819
**Family history of malignant cancer**												
Yes	308/70	178/38	0.98 (0.81-1.17)	0.803	0.99 (0.82-1.19)	0.909	213/47	30/7	0.78 (0.53-1.15)	0.213	0.81 (0.55-1.19)	0.281
No	556/148	284/82	0.94 (0.82-1.09)	0.404	0.91 (0.79-1.05)	0.187	378/103	42/12	0.81 (0.58-1.12)	0.196	0.77 (0.56-1.07)	0.118
**Histology**												
ADC	366/119	182/59	1.04 (0.87-1.24)	0.674	0.99 (0.83-1.19)	0.921	246/81	28/8	0.96 (0.65-1.43)	0.851	0.97 (0.65-1.44)	0.888
SCC	302/53	170/33	0.92 (0.76-1.11)	0.393	0.91 (0.75-1.10)	0.315	207/35	29/8	0.68 (0.46-1.01)	0.058	0.65 (0.44-0.98)	0.038
**TNM stage**												
Stage I + II	101/99	49/55	0.98 (0.70-1.38)	0.903	0.94 (0.67-1.33)	0.737	67/68	8/9	1.05 (0.50-2.18)	0.907	1.15 (0.52-2.51)	0.732
Stage III + IV	703/109	369/59	0.93 (0.82-1.06)	0.265	0.92 (0.81-1.05)	0.226	481/75	55/9	0.72 (0.54-0.96)	0.023	0.71 (0.53-0.95)	0.020

*CI, confidence interval; HR, hazard ratio; ref, reference.

a Adjusted by age, sex and hospital.

**Figure 2:**
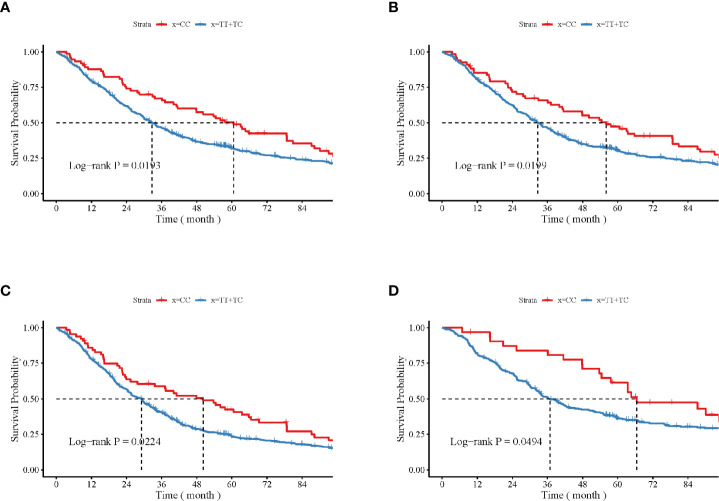
Impacts of *CAMKK1* gene polymorphism rs7214723 on the survival and prognosis of patients with lung cancer who are **(A)** male, **(B)** have smoking history, **(C)** with stage III + IV disease, and **(D)** young (aged < 60 years). The P-value for each analysis is indicated.

The impact of the *CAMKK1* gene polymorphism rs7214723 on the survival and prognosis of patients with lung cancer are shown in **Figure 2**. Among all 839 patients with available survival data, there was a trend toward longer survival in patients with the rs7214723 CC genotype who were male (*P* = 0.0193, by log-rank test), had a smoking history (*P* = 0.0199), had stage III + IV disease (*P* = 0.0224), or were young (aged < 60 years, *P* = 0.0494) when compared with those with the TT+TC genotypes (**Figure 2**). Additionally, the dominant genotypes TC+CC showed no significant association with the prognostic risk of death relative to genotype TT (**Supplementary Table S2**).

### Effects of *CAMKK1* rs7214723 on Cell Viability in A549 and NCI-H358 Cells

**Figure 3:**
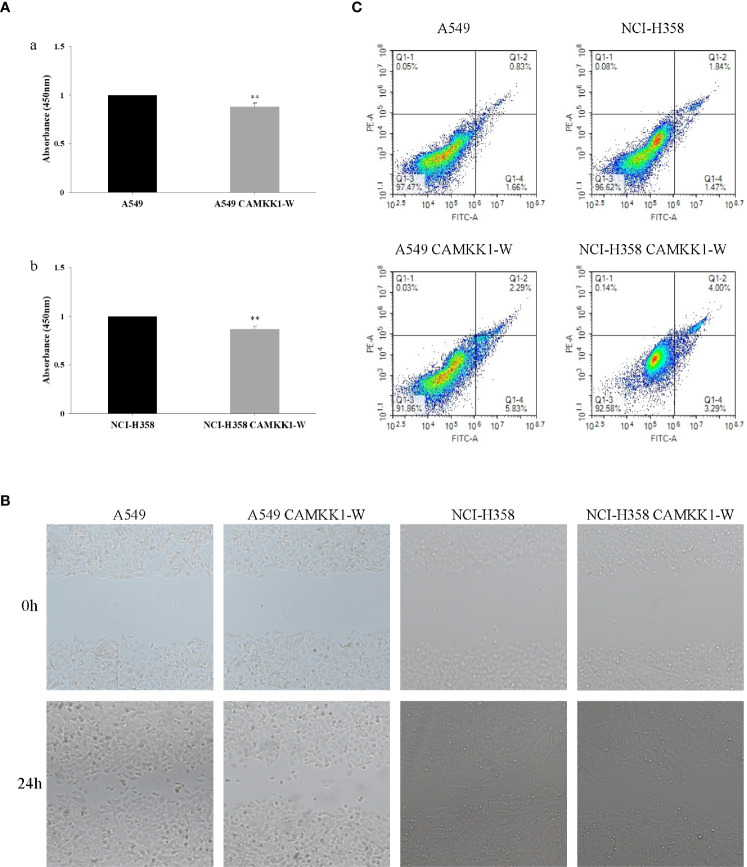
*CAMKK1* rs7214723 influences proliferation, migration and apoptosis in A549 and NCI-H358 cells. **(A)** Effects of *CAMKK1* rs7214723 on proliferation in A549 and NCI-H358 cells. All data presented the means ± SD from three independent experiments. **P < 0.01 compared with the wild-type groups. (a) Effects of *CAMKK1* rs7214723 on proliferation in A549 cells. (b) Effects of *CAMKK1* rs7214723 on proliferation in NCI-H358 cells. **(B)** Effects of *CAMKK1* rs7214723 on migration in A549 and NCI-H358 cells. **(C)** Effects of *CAMKK1* rs7214723 on apoptosis in A549 and NCI-H358 cells. Both *P* < 0.05 indicted a significant difference, compared with the wild-type groups.

The CCK-8 assay was used to assess the effects of *CAMKK1* rs7214723 on cell proliferation in A549 and NCI-H358 cells. As shown in **Figure 3**, cell growth was suppressed by *CAMKK1* rs7214723 compared with the control (both *P* < 0.05). A scratch assay was performed to measure the effect of *CAMKK1* rs7214723 on the migration of A549 and NCI-H358 cells. The result showed that liposome treatment markedly decreased the motility of A549 and NCI-H358 cells, as determined by the migration area (**Figure 3**).

### Effects of *CAMKK1* rs7214723 on Apoptosis in A549 and NCI-H358 Cells

Annexin V-FITC staining assay was performed to determine whether *CAMKK1* rs7214723 is associated with lung cancer progression, mediated by affecting the apoptosis process. A549 *CAMKK1*-w and NCI-H358 *CAMKK1*-w groups had remarkably increased apoptosis (both *P* < 0.05; **Figure 3**) when compared with the control. In summary, rs7214723 mutation of *CAMKK1* can affect the proliferation, migration, and apoptosis of A549 and NCI-H358 cells.

## Discussion

In this study, we evaluated the association between the SNP rs7214723 in *CAMKK1* and prognosis using blood samples from 839 Chinese patients with lung cancer. We found that *CAMKK1* rs7214723 T > C decreased the prognostic risk of death. Further stratified analysis showed that the risk of death significantly decreased among male patients, those aged < 60 years, those with a smoking history, those with SCC, and those with stage III + IV disease. Furthermore, CRISPR/Cas9-guided single nucleotide editing and cell functional experiment demonstrated that *CAMKK1* rs7214723 T > C significantly inhibits A549 and NCI-H358 cell proliferation and migration and promotes cell apoptosis. To the best of our knowledge, this is the first study to investigate the association between *CAMKK1* gene polymorphism rs7214723 and the prognosis of patients with lung cancer.

Previous studies have mainly focused on analyzing the association between the *CAMKK1* gene SNP rs7214723 and the risk of lung cancer. Rudd (32) conducted a large-scale genome-wide association study among British patients, analyzing 1529 cases and 2707 controls, and found that the rs7214723 (E375G) polymorphism in *CAMKK1* was a susceptibility locus of lung cancer in the UK population. Similarly, Chen (33) verified this conclusion in a study of 320 Chinese patients with lung cancer and 320 individuals in the control group, with the results showing that the rs7214723 T > C increased lung cancer risk among Chinese patients. However, no studies have reported on the association between the *CAMKK1* SNP rs7214723 and the prognosis of lung cancer. This study stratified the aforementioned analyses by epidemiological factors such as pathological type, smoking status, and disease stage and explored the impact of the loci on the prognosis of patients with lung cancer. Our results show that the SNP rs7214723 was positively correlated with the benign prognosis of lung cancer patients in the allele and recessive models.

*CAMKK1* consists of distinct N-terminal and C-terminal domains and a central kinase domain, followed by a regulatory domain consisting of overlapping autoinhibitory and calmodulin-binding domains (37). *CAMKK1* is also a serine/threonine kinase. Therefore, the expression of downstream protein kinases that it activates is significantly related to the progression of human malignancies (38). Takai et al. (12) revealed that high CaMK I and CaMK II expression in endometrial cancer is related to its increased malignant potential and that increased CaMK IV expression is significantly associated with advanced endometrial cancer (39). Similarly, CaMK I and CaMKK are involved in the control of cell cycle progression of MCF-7 human breast cancer (40). In addition, by inhibiting the CaMK IV signaling pathway, the differentiation of human neuroblastoma cells induced by retinoic acid can be accelerated (23). Studies have shown that CaMK IV can be expressed in SCLC cells, and that the inactivation of this kinase can inhibit the proliferation of SCLC (25). The activation of AMPK, which is also induced by CaMKK1, can inhibit the growth and promote the apoptosis of lung cancer cells. Accordingly, it is inferred that the activation of AMPK signaling in some tumors increases the sensitivity of certain targeted therapies (26). Our study reached a similar conclusion through CRISPR/CAS9-guided single nucleotide mutation and cell functional experiment. The results showed that *CAMKK1* rs7214723 T > C significantly inhibited the proliferation and migration of A549 and NCI-H358 cells, and inhibited cell apoptosis. This may be related to the excessive activation of *CAMKK1* and its downstream AMPK signaling pathway caused by *CAMKK1* rs7214723 T > C. These findings all prove the important role of CAMKK1 in cancer development.

The mutation SNP rs7214723 is a T to C transition resulting in the substitution of glutamate (E) to glycine (G) at the amino acid position 375. Our results indicate that rs7214723 T > C is associated with a better prognosis among patients with lung cancer, which may be due to the amino acid change in the kinase domain of CaMKK1. E375G reduces the activation ability of CaMKK1 on the downstream kinase and weakens the effect of its downstream pathway in terms of promoting the growth of lung cancer cells and inhibiting their apoptosis, which ultimately leads to a better prognosis.

In the stratified analysis, we observed that the significant association between SNP rs7214723 and the prognosis of patients with lung cancer was evident among male patients and those with history of smoking. More than 200 compounds in tobacco smoke are classified as lung carcinogens, which have been confirmed to be closely associated with increased lung cancer incidence and mortality (41, 42). Many components in tobacco smoke exert biological effects by binding the aryl hydrocarbon receptor, which is a ubiquitously expressed transcription factor (43, 44). In addition, the CaMKK/CaMK V cascade also regulates transcription by phosphorylating transcription factors such as the cAMP-response element-binding protein and the serum response factors that affect transcriptional activation (19–21). In our study, the results of the stratified analysis suggest that the *CAMKK1* gene SNP rs7214723 reduced the prognostic risk of death among patients with smoking history. We hypothesize that although the SNP rs7214723 has the effect of regulating the transcriptional activity of *CAMKK1*, its mutation has little effect. We also hypothesized that the smoking factor increases the effect of SNP rs7214723 on the function of *CAMKK1* to a certain extent, thereby delaying the progression of the lung cancer tumor, and subsequently leading to a better prognosis. Nevertheless, the specific mechanism for this remains to be clarified further. Several studies have shown that the risk of death after lung cancer diagnosis is significantly higher in men than in women (45–47). In this study, the SNP rs7214723 decreased the risk of prognostic death from lung cancer among male patients than among female patients. Smoking is a critical risk factor because men smoke more frequently than women (8, 48).

Previous reports have conducted genome-wide association studies, which can detect millions of SNPs throughout the entire genome and identify associations between SNPs and complex diseases. However, the size and complexity of the data from this method are difficult to manage, and some crucial genes could be overlooked (49). The candidate-gene associations explored in this study focused on the important locus of the *CAMKK1* gene rs7214723 to further investigate the effect on lung cancer prognosis of the SNP at this locus. We conducted a large sample size study, with a total of 839 patients with lung cancer in the survival cohort. Our results confirm that the SNP rs7214723 is associated with the prognosis and survival of patients with lung cancer, reducing the probability of false positives, improving the accuracy of the analysis, and providing a reliable estimate.

This study had some limitations. First, this result should be validated with independent studies or meta-analysis, and more extensive prospective studies are needed in the future to further evaluate the association between the *CAMKK1* SNP rs7214723 and lung cancer prognosis. Second, the subjects were of Han Chinese ethnicity, and inherent selection bias could not be completely excluded. This limits the generalization of our results to other populations. Finally, the number of SNPs analyzed in this study was limited. However, the correlation between the SNP rs7214723 and lung cancer prognosis was clear.

In conclusion, our results provided the first evidence that *CAMKK1* rs7214723 T > C is associated with poor prognosis among patients with lung cancer, playing a more significant role depending on sex, age, smoking status, histology and TNM stage. These findings have potential clinical significance for predicting the prognosis of patients with lung cancer and in the formulation of new therapeutic strategies.

## Data Availability Statement

The original contributions presented in the study are included in the article/**Supplementary Material**. Further inquiries can be directed to the corresponding authors.

## Ethics Statement

The studies involving human participants were reviewed and approved by the ethics committee of the School of Life Sciences, Fudan University. The patients/participants provided their written informed consent to participate in this study.

## Author Contributions

Conception and design: all authors. Administrative support: LJ, MX, FL, and JJW. Provision of study materials or patients: JF, YL, MX, FL, and JJW. Collection and assembly of data: HZ, ZZ, YL, XH, and CX. Cell functional experiments: BC, ZZ, and MX. Data analysis and interpretation: HZ and YL. Manuscript writing: HZ, ZZ, and JJW. Final approval of manuscript: all authors. All authors agree to be accountable for the content of the work. All authors contributed to the article and approved the submitted version.

## Funding

This work was supported by Shanghai Committee of Science and Technology (grant number 20Z11901002), National Natural Science Foundation of China (grant number 81372236, 81360359, and 82160847), and Key Project of the National Science and Technology Pillar Program (grant number 2011**BAI**09**B**00).

## Conflict of Interest

The authors declare that the research was conducted in the absence of any commercial or financial relationships that could be construed as a potential conflict of interest.

## Publisher’s Note

All claims expressed in this article are solely those of the authors and do not necessarily represent those of their affiliated organizations, or those of the publisher, the editors and the reviewers. Any product that may be evaluated in this article, or claim that may be made by its manufacturer, is not guaranteed or endorsed by the publisher.

## Abbreviations

AMPK, 5’-AMP-activated protein kinase; ASC, adenosquamous carcinoma; CaMK, Ca^2+^/calmodulin-dependent protein kinase; CaMKK, Ca2+/calmodulin-dependent protein kinase kinase; CI, confidence interval; CS, carcinosarcoma; HCC, hepatocellular carcinoma; HR, adjusted hazard ratio; HWE, Hardy-Weinberg equilibrium; LCC, large cell carcinoma; MEC, mucoepidermoid carcinoma; MST, median survival time; SCC, squamous cell carcinoma; SCLC, small cell lung cancer; SNP, single nucleotide polymorphism.
