# Supplementary material for: Associations Between CAMKK1 Polymorphism rs7214723 and the Prognosis of Patients With Lung Cancer
Source: Front Oncol. 2021 Nov 19;11:757484. doi: 10.3389/fonc.2021.757484 (PMC8640188; doi:10.3389/fonc.2021.757484)
Supplement: Supplementary file 1 [file Table_1.docx]

| **Variables** | **Site 1**^#^ | | |  | **Site 2^&^** | | |
| --- | --- | --- | --- | --- | --- | --- | --- |
|  | **N (%)** | **MST^£^** | ***P*** |  | **N (%)** | **MST** | ***P*** |
| **All** | 507 | 30.37 |  |  | 332 | 41.07 |  |
| **Gender** |  |  | 0.058 |  |  |  | 0.038 |
| Female | 148 (29.2%) | 37.73 |  |  | 81 (24.4%) | 42.63 |  |
| Male | 359 (70.8%) | 26.9 |  |  | 251 (75.6%) | 40.03 |  |
| **Age** |  |  | 0.039 |  |  |  | 0.017 |
| Age < 60 | 213 (42.0%) | 39.1 |  |  | 102 (30.7%) | 47.87 |  |
| Age ≥ 60 | 294 (58.0%) | 26.73 |  |  | 230 (69.3%) | 38.5 |  |
| **Smoking status** |  |  | 0.004 |  |  |  | 0.059 |
| Nonsmoker | 154 (30.4%) | 38.8 |  |  | 83 (25.0%) | 43.93 |  |
| Smoker | 333 (65.7%) | 25.93 |  |  | 249(75.0%） | 40 |  |
| Unknown | 20 (3.9%) | 67 |  |  | 0 |  |  |
| **Family history of malignant cancer** |  |  | 0.182 |  |  |  | 0.689 |
| Yes | 163 (32.1%) | 22.83 |  |  | 139 41.9%） | 42.53 |  |
| No | 344 (67.9%) | 36.17 |  |  | 193 (58.1%) | 40.03 |  |
| **Histology** |  |  | 0.253 |  |  |  | 0.524 |
| ADC | 259 (51.1%) | 36.83 |  |  | 108 (32.5%) | 45.23 |  |
| SCC | 147 (29.0%) | 26.73 |  |  | 135 40.7%） | 38.5 |  |
| SCLC | 35 (6.9%) | 25.63 |  |  | 37 (11.1%) | 36.9 |  |
| Others* | 66 (13.0%) | 19.87 |  |  | 52 (15.7%) | 42.63 |  |
| **TNM Stage** |  |  | < 0.001 |  |  |  | < 0.001 |
| Stage Ⅰ + Ⅱ | 120 (23.7%) | 114.13 |  |  | 34 (10.2%) | 110.43 |  |
| Stage III + Ⅳ | 377 (74.4%) | 20.17 |  |  | 248 (74.7%) | 37.17 |  |
| Unknown | 10 (2.0%) | 19.27 |  |  | 50 (15.1%) | 66.43 |  |

**Supplementary Tables**

**Table S1.** Distribution of characteristics in Chinese patients with lung cancer and prognosis analysis

* Other carcinomas include ASC, LCC, CS, MEC.

^#^ Site 1, Changhai Hospital affiliated with the Naval Military Medical University.

**^&^**Site 2, Taizhou Institute of Health Sciences, Fudan University.

**^£^** MST, median survival time.

**Table S2.** Association between *CAMKK1* polymorphisms in dominant model and prognosis in Chinese patients with lung cancer

| **Stratification** | **Allele model** | | | | | |  | **Dominant model** | | | | | |
| --- | --- | --- | --- | --- | --- | --- | --- | --- | --- | --- | --- | --- | --- |
|  | **Death/survive** | | **HR (95% CI)*** | ***P*** | **HR^a^ (95% CI)** | ***P*^a^** |  | **Death/survive** | | **HR (95% CI)** | ***P*** | **HR^a^ (95% CI)** | ***P*^a^** |
|  | **T(ref)** | **C** |  |  |  |  |  | **T/T (ref)** | **T/C+C/C** |  |  |  |  |
| **Gender** |  |  |  |  |  |  |  |  |  |  |  |  |  |
| Male | 639/131 | 355/83 | 0.87(0.77-0.99) | 0.040 | 0.88(0.77-1.00) | 0.054 |  | 200/40 | 297/67 | 0.90(0.75-1.07) | 0.235 | 0.91(0.76-1.09) | 0.285 |
| Female | 225/87 | 107/37 | 1.15(0.91-1.45) | 0.246 | 1.13(0.89-1.42) | 0.315 |  | 73/28 | 93/34 | 1.25(0.91-1.70) | 0.165 | 1.22(0.89-1.67) | 0.213 |
| **Age (year)** |  |  |  |  |  |  |  |  |  |  |  |  |  |
| ≥ 60 | 565/110 | 301/64 | 0.95(0.83-1.10) | 0.499 | 0.96(0.84-1.11) | 0.592 |  | 182/32 | 251/55 | 0.97(0.80-1.17) | 0.733 | 0.98(0.81-1.19) | 0.856 |
| < 60 | 299/108 | 161/56 | 0.95(0.78-1.15) | 0.611 | 0.90(0.74-1.10) | 0.318 |  | 91/36 | 139/46 | 1.09(0.84-1.43) | 0.509 | 1.04(0.78-1.37) | 0.807 |
| **Smoking status** |  |  |  |  |  |  |  |  |  |  |  |  |  |
| Yes | 623/116 | 341/74 | 0.87(0.76-0.99) | 0.037 | 0.88(0.77-1.00) | 0.054 |  | 195/35 | 287/60 | 0.89(0.74-1.07) | 0.205 | 0.90(0.75-1.08) | 0.255 |
| No | 224/93 | 114/39 | 1.15(0.92-1.44) | 0.231 | 1.12(0.89-1.41) | 0.332 |  | 72/29 | 97/37 | 1.23(0.91-1.68) | 0.183 | 1.20(0.88-1.63) | 0.252 |
| **Family history of malignant cancer** |  |  |  |  |  |  |  |  |  |  |  |  |  |
| Yes | 308/70 | 178/38 | 0.98(0.81-1.17) | 0.803 | 0.99(0.82-1.19) | 0.909 |  | 95/23 | 148/31 | 1.08(0.83-1.39) | 0.574 | 1.09(0.84-1.41) | 0.534 |
| No | 556/148 | 284/82 | 0.94(0.82-1.09) | 0.404 | 0.91(0.79-1.05) | 0.187 |  | 178/45 | 242/70 | 0.97(0.80-1.18) | 0.767 | 0.92(0.76-1.12) | 0.434 |
| **Histology** |  |  |  |  |  |  |  |  |  |  |  |  |  |
| ADC | 366/119 | 182/59 | 1.04(0.87-1.24) | 0.674 | 0.99(0.83-1.19) | 0.921 |  | 120/38 | 154/51 | 1.09(0.85-1.38) | 0.499 | 0.99(0.78-1.27) | 0.962 |
| SCC | 302/53 | 170/33 | 0.92(0.76-1.11) | 0.393 | 0.91(0.75-1.10) | 0.315 |  | 95/18 | 141/25 | 1.03(0.79-1.34) | 0.813 | 1.03(0.78-1.34) | 0.846 |
| **TNM stage** |  |  |  |  |  |  |  |  |  |  |  |  |  |
| Stage Ⅰ + Ⅱ | 101/99 | 49/55 | 0.98(0.70-1.38) | 0.903 | 0.94(0.67-1.33) | 0.737 |  | 34/31 | 41/46 | 0.95(0.60-1.49) | 0.814 | 0.86(0.54-1.36) | 0.510 |
| Stage III + Ⅳ | 703/109 | 369/59 | 0.93(0.82-1.06) | 0.265 | 0.92(0.81-1.05) | 0.226 |  | 222/34 | 314/50 | 1.00(0.84-1.19) | 0.976 | 0.99(0.83-1.18) | 0.947 |

* CI, confidence interval; HR, hazard ratio; ref, reference. ^a^ Adjusted by age, sex and hospital.
